# Management of Wernicke's encephalopathy in a pregnant woman at 27 weeks gestation complicated by pre-eclampsia: A case report

**DOI:** 10.1016/j.radcr.2024.12.018

**Published:** 2025-01-03

**Authors:** Amine Bensaid, Marouane Boukroute, Safae Bekkaoui, Hajar Berrichi, Abdelilah Elrhalete, Hamza Mimouni, Younes Oujidi, Houssam Bkiyar, Brahim Housni, Ahmed Mimouni

**Affiliations:** a Intensive Care Unit, Mohammed VI University Hospital Center, Faculty of Medicine and Pharmacy, Oujda, Morocco; b Department of Obstetrics and Gynecology, Mohammed VI University Hospital Center, Faculty of Medecine and Pharmacy, Oujda, Morocco; c Mohammed First University Oujda, Oujda, Morocco

**Keywords:** Thiamine deficiency, Wernicke's encephalopathy, emergency MRI, Pre-eclampsia

## Abstract

Wernicke's Encephalopathy (WE) is a rare but severe condition primarily caused by thiamine deficiency, often seen in pregnant women who experience severe vomiting, such as in hyperemesis gravidarum. This case report details a 38-year-old woman at 27 weeks of gestation who developed altered consciousness, cerebellar ataxia, and hyperlactatemia following persistent vomiting. Brain MRI demonstrated characteristic bilateral abnormalities consistent with WE. Early recognition and prompt intravenous thiamine administration led to significant clinical improvement. This case emphasizes the importance of maintaining a high level of suspicion and initiating immediate treatment to prevent irreversible neurological damage in pregnant women presenting with severe vomiting and neurological symptoms. A unique feature of this case is the development of Wernicke's encephalopathy secondary to hyperemesis gravidarum, further complicated by severe pre-eclampsia.

## Introduction

Wernicke's encephalopathy (WE) is a rare neurological disorder, firts described by Carl Wernicke in 1881, it is due to thiamine deficiency, a water-soluble vitamin essential for carbohydrate metabolism, observed in approximately 60% of cases [1] of patients presenting with the triad of ocular signs, ataxia, and confusion.

We present a case of Wernicke's encephalopathy (WE) associated with hyperemesis gravidarum and hyperlactatemia. This case highlights the critical importance of maintaining a high index of suspicion for WE and initiating prompt treatment in pregnant women presenting with severe vomiting and neurological symptoms [2].

## Case report

A 38-year-old woman, G1P0, at 27 weeks of gestation, with a history of laparoscopic cholecystectomy 10 years prior and an unremarkable postoperative course, presented with no significant family history. She was admitted to the intensive care unit for management of altered consciousness, which was preceded by severe epigastric pain and intractable vomiting lasting for over a month. Physical examination revealed a confused patient with a Glasgow Coma Scale score of 13/15, agitation, cerebellar ataxia, nystagmus, and paresthesia in both the upper and lower limbs. Her blood pressure was 165/90 mmHg, heart rate 130 bpm, and respiratory rate 30 breaths per minute. Urinalysis demonstrated 3+ proteinuria.

The laboratory findings revealed several significant abnormalities. The hemoglobin level was 11.3 g/dL (normal range: 12.0-16.0 g/dL). The platelet count was within normal limits at 182,000/μL (normal range: 150,000-450,000/μL). However, both aspartate aminotransferase (ASAT) and alanine aminotransferase (ALAT) were markedly elevated. ASAT was 8 times above the normal range, while ALAT was 4 times elevated (normal range: ASAT 10-40 U/L, ALAT 7-56 U/L). The prothrombin time was reduced to 55%, below the normal range of 70%-100%. Potassium levels were found to be low at 3.2 mmol/L (normal range: 3.5-5.0 mmol/L), and creatinine was significantly elevated at 10.82 mg/dL (normal range: 0.6-1.2 mg/dL). Urea levels were 0.43 g/dL (normal range: 0.7-1.2 g/dL). Arterial blood gas analysis revealed a pH of 7.54, indicative of metabolic alkalosis (normal range: 7.35-7.45). The partial pressure of oxygen (PaO2) was elevated at 148 mmHg, exceeding the normal range of 75-100 mmHg. The partial pressure of carbon dioxide (PaCO2) was low at 6.1 mmHg (normal range: 35-45 mmHg), suggesting respiratory alkalosis. The bicarbonate (HCO3-) level was low at 6.1 mmol/L (normal range: 22-28 mmol/L), and lactate levels were elevated at 7.9 mmol/L, indicating hyperlactatemia (normal range: 0.5-2.2 mmol/L). Additionally, proteinuria was present, with a level of 900 mg/mL, significantly above the normal range of <150 mg/mL.

**Fig. 1:**
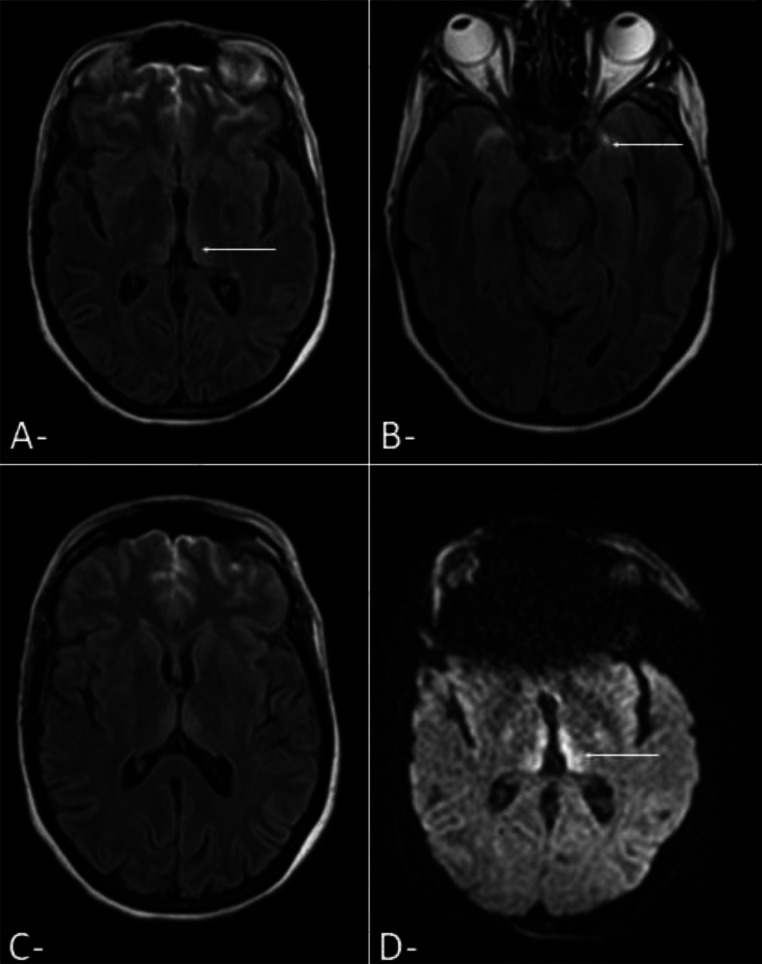
Emergency MRI images in axial cross-section T2 (A-C) and diffusion (D) revealing bilateral symmetrical cortical hypersignal in the dorso-medial thalami, peri-aqueductal regions, and mammillary bodies.

An emergency cesarean section was performed due to the patient's clinical deterioration, with no improvement despite supportive management. This prompted the decision to conduct a contrast-enhanced brain CT scan to investigate potential findings that could explain the clinical presentation. The CT scan results were normal. Given the persistence of neurological symptoms, including nystagmus, cerebellar ataxia, and paresthesia, a brain MRI was subsequently performed (Fig. 1). The MRI revealed bilateral, symmetrical signal abnormalities in the dorso-medial thalami, peri-aqueductal regions, and mammillary bodies. Based on significantly low levels of Vitamin B1 (thiamine), Wernicke's encephalopathy was diagnosed.

The treatment regimen consisted of parenteral thiamine administration, with doses ranging from 200 to 500 mg 3 times daily for 3-5 days, followed by 250-1000 mg daily orally. Follow-up included daily laboratory tests, including an electrolyte panel, liver function tests, and a complete blood count, as well as periodic arterial blood gas measurements. The patient also underwent physiotherapy, which contributed to significant clinical and biochemical improvement, including the normalization of lactate levels. She was deemed fit for discharge 5 days later.

A distinctive feature of this case is the development of Gayet-Wernicke syndrome secondary to hyperemesis gravidarum, further complicated by severe pre-eclampsia.

## Discussion

Wernicke's encephalopathy (WE) is most commonly diagnosed in individuals with alcohol use disorder, with a prevalence of approximately 12.5%. In contrast, the prevalence of WE in nonalcoholic individuals ranges from 0.04% to 0.13% [3]. Wernicke's encephalopathy is a neuropsychiatric syndrome caused by thiamine deficiency, a water-soluble vitamin crucial for carbohydrate metabolism. In instances of acute thiamine deficiency, pyruvate accumulates and is converted into lactate. Chronic deficiency may result in polyneuropathy and the subsequent onset of Wernicke's encephalopathy [4]. This explains hyperlactatemia in our case report. Wernicke's encephalopathy (WE) can be precipitated by factors such as vomiting, malnutrition, and various other conditions. It is typically characterized by persistent vomiting during pregnancy without any other identifiable cause. The link between WE and hyperemesis gravidarum was first reported in 1914 [3]. Hyperemesis gravidarum is a preventable risk factor for nonalcoholic Wernicke's encephalopathy (GWE). Due to the increased thiamine requirements during pregnancy, symptoms may emerge as early as 6 weeks of gestation and can progress rapidly [5].

Classic symptoms of Wernicke's encephalopathy include alterations in mental status, ophthalmoplegia, and ataxia; however, these symptoms are only observed in a minority of patients [6]. Additional symptoms that may manifest alongside or instead of the classic triad include hypothermia, hypotension, and coma [7]. In retrospective [8] and prospective [9,10] studies, thiamine deficiency has been reported to have a significant prevalence in both adult (10%-20%) and pediatric (28%) populations. It can become clinically evident in any form of malnutrition that depletes the body's thiamine reserves, which typically last 2-3 weeks. Conditions such as alcoholism, bariatric surgery, and hyperemesis gravidarum are among those that can lead to thiamine deficiency [6].

Previous studies have suggested that brain imaging techniques, including magnetic resonance imaging (MRI), may be advantageous for the early diagnosis of acute Wernicke's encephalopathy [11,12]. With a sensitivity of 93% [13], MRI remains the gold standard for WE diagnosis.

Literature has suggested that brain imaging techniques, including magnetic resonance imaging (MRI), may be advantageous for the early diagnosis of acute Wernicke's encephalopathy [11,12]. Although CT scans may reveal hypodense areas in the mammillary bodies and periventricular regions, these findings are often subtle. In contrast, MRI provides superior sensitivity and specificity for diagnosing Wernicke's encephalopathy, with sensitivity approaching 92% and specificity near 100%. MRI detects hyperintensities on T2-weighted and FLAIR sequences in the mammillary bodies, periventricular regions, and thalamus, and can also identify changes in the basal ganglia and adjacent ventricular areas. Overall, MRI is preferred due to its enhanced ability to detect subtle tissue abnormalities, making it a more reliable imaging modality than CT [14].

Gayet-Wernicke encephalopathy (GWE) is a medical emergency that necessitates the immediate administration of intravenous thiamine upon diagnosis to prevent irreversible neurological sequelae. Thiamine supplementation should be initiated as soon as Wernicke's encephalopathy is suspected. The European Federation of Neurological Societies (EFNS) guidelines recommend administering 200 mg of intravenous thiamine 3 times daily, starting before any carbohydrate intake, and continuing until there is no further improvement in signs and symptoms [15]. In nonalcoholic patients, an intravenous dose of thiamine 100-200 mg once daily could be enough; whereas in alcoholic patients, higher doses may be required [13]. Evolution can include the full reversibility of disorders, motor sequelae, Korsakoff's syndrome, coma and even death; the mortality rate ranges from 20% to 30% [16].

## Conclusion

Wernicke's encephalopathy is an uncommon condition during pregnancy that should be considered in any neurological disorder arising in the context of inadequate nutritional status, particularly in cases of hyperemesis gravidarum. Prompt intervention with vitamin supplementation (thiamine) can lead to a favorable outcome and prevent the persistence of neurological symptoms. Delayed diagnosis or treatment, however, may result in irreversible neurological sequelae or even death.

## Patient consent

The patient gave her informed consent for this case report to be published.
